# Neuro-immune crosstalk in breast cancer: molecular mechanisms from stress signals to immune escape

**DOI:** 10.3389/fimmu.2026.1790195

**Published:** 2026-03-13

**Authors:** Gaofeng Ni, Nuan Qian, Ying Jiang, Rui Tian, Tianjiao Huang, Shuyan Li

**Affiliations:** 1 Department of Breast Surgery, Yantaishan Hospital Affiliated to Binzhou Medical University, Yantai, China; 2 School of Life Sciences and Technology, Henan Medical University, Xinxiang, China; 3 Shandong College of Traditional Chinese Medicine, Yantai, China; 4 The First School of Clinical Medicine, Heilongjiang University of Traditional Chinese Medicine, Harbin, China

**Keywords:** breast cancer, circadian rhythms, innervation, neuro-immune interaction, parasympathetic nerve, sympathetic nerve

## Abstract

**Background:**

Breast cancer (BC) exhibits marked biological heterogeneity and remains a major cause of cancer-related death in women. With advances in molecular subtyping and tumor microenvironment research, the nervous system has emerged as an important regulator of BC initiation, progression, and metastasis. Tumor-associated nerves influence cancer not only locally through sympathetic, parasympathetic, and sensory innervation, but also systemically by modulating immunity via psychological stress, circadian rhythms, and neuroendocrine pathways.

**Results:**

Increasing evidence shows that nerve fiber density is elevated in BC tissues and correlates with greater invasiveness and poorer prognosis. The sympathetic nervous system promotes tumor growth, angiogenesis, and metastasis mainly through β-adrenergic signaling and suppression of anti-tumor immunity. Chronic psychological stress further enhances tumor-promoting immune changes through sustained neuroendocrine activation. In contrast, parasympathetic signaling may exert inhibitory effects on tumor progression, while sensory nerves and neuropeptides display context-dependent roles in inflammation, pain, and immune regulation. Moreover, circadian rhythm disruption and reduced melatonin impair tumor immune surveillance. Clinical and retrospective studies suggest that neural-targeted interventions, such as β-blockers and psychological therapies, may improve the immune microenvironment and clinical outcomes in BC.

**Conclusion:**

The nervous system plays a crucial role in shaping the BC microenvironment and promoting immune escape through complex, multi-level mechanisms. Different types of nerves exert distinct effects on tumor progression. Targeting neuro-immune interactions may therefore offer a promising strategy for adjuvant BC therapy.

## Graphical Abstract

**Figure:**
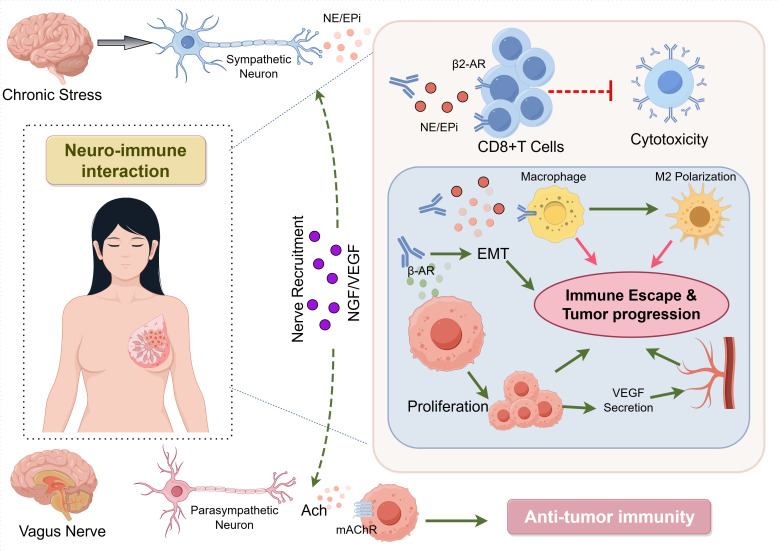
A schematic diagram of the interaction between the nervous system, tumors and immune cells in BC.

## 1 Introduction

Breast cancer (BC) is among the most prevalent malignancies affecting women worldwide (1). Despite the continuous improvement of early screening methods and the increasingly refined comprehensive treatment model, the emergence of tumor recurrence, distant metastasis and drug resistance still seriously affects the long-term survival of BC patients (2). Increasing evidence suggests that the biological complexity of BC cannot be explained solely by tumor cell–intrinsic factors. Beyond immune cells, fibroblasts, and vasculature, tumor-associated nerves are now recognized as an important yet previously overlooked component of the tumor microenvironment (3).

Recent advances in multi-omics technologies and machine learning algorithms have enabled systematic identification of key oncogenic drivers across various cancer types. For instance, a study utilized single-cell sequencing, spatial transcriptomics, and multiple machine learning approaches to identify PAK2 as a central regulator of programmed cell death and cancer stem cell properties in head and neck squamous cell carcinoma. Such integrative frameworks demonstrate the power of systems biology in decoding complex tumorigenic networks and provide valuable methodological references for studying neuro-immune interactions in BC (4).

In previous studies, the role of the nervous system in tumors has mostly been regarded as an accessory process of pain conduction or endocrine regulation. However, recent evidence shows that significant neural structural and functional remodeling occurs within tumor tissues. Cancer cells can attract nerve fibers into the tumor area and enable them to participate in the regulation of the local microenvironment. The increase in nerve fiber density in BC is often closely related to enhanced tumor invasion ability, intensified immunosuppression and poor prognosis. This indicates that the nervous system is not a bystander but plays an active and crucial regulatory role in the process of tumor progression.

Distinct nerve types exert divergent effects in BC. Sympathetic nerves promote tumor growth, angiogenesis, and metastasis through the release of norepinephrine and epinephrine (NE/EPi), while concurrently suppressing effector immune cell function (5, 6). In contrast, parasympathetic nerves have been reported to limit tumor invasion and dissemination in several studies (7), whereas sensory nerves and their neuropeptide signals exhibit pronounced context-dependent effects on inflammation, pain, and immune modulation (8).

Beyond local innervation, systemic neural regulation also influences BC progression. Chronic psychological stress and circadian rhythm disruption impair tumor immune surveillance via the neuroendocrine-immune network (9, 10). Against this backdrop, the present review summarizes the characteristics of neural innervation in BC, elucidates its immunoregulatory effects, and discusses the potential implications for disease progression and therapeutic intervention.

## 2 Neuro-immune regulation and clinical implications in BC

### 2.1 Remodeling of the BC microenvironment: neural regulation in tumor initiation and progression

Compared with normal breast tissue or benign lesions, the distribution of nerve fibers in BC tissues is significantly increased, and the increase in the degree of nerve innervation is often closely related to the enhanced invasiveness of the tumor and adverse clinical outcomes (11). The so-called tumor neurogenesis refers to the process in which tumors actively attract peripheral nerves to grow towards the tumor area by secreting various neurotrophic factors, thereby inducing axon growth in the tumor microenvironment (12, 13). In BC, this process predominantly originates from peripheral nerves and is closely associated with tumor-derived nerve growth factor (NGF) and vascular endothelial growth factor (VEGF) (14, 15).

Different types of nerves have significantly different effects on the progression of BC. Clinical and pathological studies have found that an increase in sympathetic nerve density is often associated with an increased risk of tumor recurrence and a shortened survival period, while a higher density of parasympathetic nerves shows the opposite trend (16). In recurrent BC lesions, the neural distribution pattern, characterized by enhanced sympathetic nerves and reduced parasympathetic nerves, can often be observed. This suggests that the distribution pattern of nerves may have certain prognostic value in itself.

The neural networks within breast tissue not only perform sensory and autonomic functions, but also directly participate in the occurrence and progression of tumors by regulating local immune responses, tissue remodeling, and angiogenesis, among other processes (17). Concepts such as tumor innervation, perineural invasion, and nerve-cancer cell crosstalk have gained increasing attention, emphasizing that the nervous system constitutes an active regulatory component of tumor biology (18). Nowadays, there is growing evidence suggesting that there is not a one-way influence between cancer cells and neurons, but rather a mutual exchange of signals. For instance, neurotransmitters such as norepinephrine, dopamine, and serotonin can activate signaling pathways that promote tumor growth, help blood vessels form, and suppress immune responses. Conversely, cancer cells also affect the functions and survival of neurons, thereby reshaping the tumor microenvironment (19, 20).

At the immune level, whether the neural structure is intact is closely related to the body’s anti-tumor immune capacity (21). Research has found that when nerves are damaged, the infiltration level of CD8^+^ T cells in tumor tissues often drops significantly, suggesting that once the neuro-immune axis is disrupted, it may provide conditions for tumors to achieve immune escape by weakening the cytotoxic effects mediated by CD8^+^ T cells (such as the release of granzyme B) (22, 23). Accordingly, targeting neuro-immune interactions may represent a novel therapeutic avenue in BC.

### 2.2 Sympathetic nerves and adrenergic signaling: drivers of BC progression

#### 2.2.1 β-adrenergic receptor activation: induction of macrophage M2 polarization and angiogenesis

By regulating macrophage polarization toward the M2 phenotype and activating the β-catenin signaling pathway, an immunomodulatory and repair-supportive microenvironment can be established, which is beneficial for tissue remodeling and peripheral nerve regeneration. Interestingly, similar neuro-immune regulatory mechanisms are also involved in tumor progression (24).

The continuous and excessive activation of the sympathetic nervous system is widely regarded as one of the important factors driving the progression of BC. Sympathetic nerve terminals within tumors continuously release norepinephrine and epinephrine, which act on α- and β-adrenergic receptors expressed on tumor cells and immune cells, thereby promoting tumor cell proliferation, invasion, migration, and distant metastasis. In addition, norepinephrine exhibits chemotactic activity, enhancing the recruitment of tumor-associated cells and modulating their migratory behavior (25).

At the level of immune regulation, sympathetic nerve signaling mainly suppresses anti-tumor immune responses through β_2_-adrenergic receptors, in a tumor microenvironment of BC that is already characterized by abundant infiltration of macrophages with an M2-like phenotype (26). Although many tumors contain CD8^+^ T cell infiltration (27), activation of the noradrenaline/β-adrenergic pathway promotes polarization of tumor-associated macrophages toward an immunosuppressive M2 phenotype. It also inhibits CD8^+^ T-cell proliferation and interferon-γ secretion (28), reduces natural killer cell activity, and weakens Th1-type immune responses. Collectively, these effects impair antitumor immune surveillance (29, 30). At the same time, this pathway can also promote the accumulation of myeloid-derived suppressor cells and regulatory T cells, further strengthening the immunosuppressive state in the tumor microenvironment (31, 32).

In addition to its inhibitory effect on immune responses, β-adrenergic signaling can also promote the expression of various angiogenic factors, thereby facilitating the formation of new blood vessels in tumors and creating a favorable microenvironment for tumor growth and metastasis. In BC-related models, long-term exposure to stress can lead to a continuous increase in norepinephrine and epinephrine levels, thereby significantly accelerating tumor development and metastasis. This further indicates that the sympathetic nerve-β-adrenergic pathway plays a core role in tumor angiogenesis and immune escape processes.

#### 2.2.2 Stress-response pathways: synergistic actions of the HPA axis and local sympathetic innervation in metastasis

Long-term psychological stress states (such as anxiety and depression) can simultaneously activate the hypothalamic-pituitary-adrenal (HPA) axis and the sympathetic nervous system, promoting the progression of BC at both the systemic and local levels (33). Under continuous stress conditions, the continuous release of glucocorticoids and catecholamines will extensively inhibit the function of immune cells and weaken the anti-tumor immune response mediated by CD8^+^ T cells, thereby accelerating tumor growth and lymphatic metastasis (9).

In recent years, studies have shown that the gut microbiota plays a crucial regulatory role and is regarded as an important factor connecting stress, the immune system and tumor progression. Under chronic stress conditions, the balance of the intestinal microecology is often disrupted, prominently manifested as a reduction in beneficial bacterial communities such as *Blautia* and *Akkermansia muciniphila*, as well as a decrease in the production of short-chain fatty acids like acetic acid and butyric acid. This imbalance suppresses CD8^+^ T cell activity, thereby impairing anti-tumor immunity and facilitating tumor progression and metastasis. It is notable that the disruption of the Blautia-acetic acid axis has been considered an important mechanistic link between depressive state and accelerated progression of BC (34). Collectively, sympathetic activation drives BC progression through β-adrenergic signaling that integrates three major effects: direct tumor cell stimulation, promotion of angiogenesis, and suppression of antitumor immunity. This coordinated neuro-immune regulation provides a mechanistic link between chronic stress and tumor metastasis. Although the β-adrenergic signal mainly mediates local tumor immune interactions, the activation of the HPA axis enables this effect to manifest throughout the body. Under chronic stress conditions, continuous release of glucocorticoids will alter the distribution and function of immune cells outside the tumor site, thereby achieving an immunosuppressive state.

### 2.3 Sensory and parasympathetic nerves: emerging modulators of the BC microenvironment

#### 2.3.1 Alterations in nociceptive signaling: a malignant feedback loop between pain and tumor-associated inflammation

The sensory nerves, especially the neurons expressing transient receptor potential vanilloid 1 (TRPV1), are widely present in the BC microenvironment and can participate in tumor-related inflammatory responses and immune regulation by releasing various neuropeptides. The bidirectional interaction between tumor cells and sensory nerves is not only an important basis for the generation of cancer pain, but also affects the occurrence, development, and metastasis of tumors to a certain extent.

Neurotransmitters such as calcitonin gene-related peptide (CGRP) and substance P, by binding to G protein-coupled receptors on immune cells, can trigger inflammatory responses and influence the direction of immune polarization (35). Existing studies have shown that the CGRP signaling pathway can inhibit the function of effector immune cells, thereby providing conditions for tumor immune escape. Intervention targeting the CGRP-RAMP1 axis not only alleviates tumor-related pain but also enhances the anti-tumor immune response to a certain extent (36). In contrast, substance P can induce mast cell degranulation by activating the neurokinin-1 receptor, increase vascular permeability, and promote the infiltration of white blood cells into the tumor site, thereby creating an inflammatory microenvironment conducive to tumor growth. At the level of immune regulation, substance P can stimulate mast cells to release histamine, enhance the cytotoxic activity of natural killer cells, and participate in vasodilation by inducing the release of nitric oxide, playing an important role in neurogenic inflammatory responses (37). Notably, substance P can also directly act on the receptors on the surface of tumor cells, promoting the metastasis of BC cells and making it a potential therapeutic target (38). The synthesis, release, and actions of these neuropeptides are highly complex, reflecting the exquisite precision of neuroimmune regulation.

The role of sensory nerves in the progression of BC is not fixed but rather dynamically adjusts according to the tumor stage, the degree of local inflammation, and the composition of infiltrating immune cells. Taking a highly inflammatory microenvironment as an example, if there are abundant mast cells and M2-type macrophages, neuropeptides such as substance P and other signaling molecules can further stimulate tumor-promoting inflammation and enhance vascular permeability, thereby promoting tumor growth and spread (8). Conversely, when cytotoxic lymphocytes still maintain functional activity, sensory nerve signals are believed to help maintain the anti-tumor activity of CD8^+^ T cells and natural killer cells, thereby exerting a certain inhibition on the metastasis and colonization of distant organs. Thus, whether sensory nerves exert a promoting or inhibitory effect on BC mainly depends on the balance between the inflammatory amplification signals in the tumor microenvironment and effective anti-tumor immunity (35).

#### 2.3.2 Loss of the cholinergic anti-inflammatory pathway: tumor-suppressive potential of the parasympathetic nervous system

In contrast to the tumor-promoting effects of sympathetic activation, a growing body of evidence suggests that parasympathetic nervous system activity may exert inhibitory effects on BC progression (11). In multiple *in vivo* models, stimulation of parasympathetic nerves significantly delays breast tumor growth, whereas vagotomy promotes distant metastasis, indicating a protective role of parasympathetic signaling in constraining tumor invasion and dissemination (39). Nevertheless, cholinergic signaling does not operate unidirectionally. Aberrant or excessive activation of cholinergic receptors, particularly muscarinic acetylcholine receptors, may paradoxically promote BC progression under specific conditions (40). This observation suggests that the tumor-suppressive effects of parasympathetic nerves likely arise from integrated regulation of tumor cells and diverse cellular components within the tumor microenvironment, rather than from a single linear signaling pathway.

Acetylcholine serves as the primary neurotransmitter of the parasympathetic nervous system and is a key mediator of cholinergic regulation within the immune system. It should be pointed out that the current evidence regarding the specific mechanism of cholinergic signaling in anti-tumor immunity mainly comes from non-BC tumor models. Evidence from both clinical studies and experimental animal models indicates that signaling through muscarinic receptors influences interactions between antigen-presenting cells and T lymphocytes, promotes metabolic and functional activation of T cells, and controls the recruitment of regulatory T cells to peripheral tissues (41). In several non-BC models, surgical vagotomy has been shown to accelerate tumor growth and invasive behavior, whereas targeted modulation of muscarinic pathways reshapes immune cell infiltration and limits tumor progression (42). In addition, cholinergic input from the vagus nerve can restrain inflammatory responses and tumor development by modulating memory T-cell function (42, 43). In the field of BC, current research mainly focuses on the expression profile analysis of cholinergic receptors in clinical samples and their association with prognosis, as well as the functional verification of receptors based on BC cell lines. However, the direct evidence regarding the mechanism by which it regulates the immune response to affect tumor progression is still relatively limited. Therefore, the above findings from non-BC models provide important references for understanding the tumor-suppressing mechanism of parasympathetic signals in BC, but further verification is still needed in BC-specific models and clinical cohorts.

### 2.4 Circadian rhythm and endocrine disruption: systemic regulatory challenges

#### 2.4.1 Circadian dysregulation and immune surveillance in BC: synergy between melatonin and neuroendocrine pathways

The circadian rhythm is an evolutionarily conserved timekeeping system within living organisms, responsible for coordinating environmental signals such as light with internal processes like metabolism, endocrine, and immunity, thereby maintaining physiological homeostasis (43–45). A growing body of epidemiological and experimental evidence links circadian disruption to increased BC risk and progression, particularly among individuals engaged in long-term night-shift work or irregular schedules (46, 47).

Perturbation of circadian rhythms undermines tumor immune surveillance largely through neuroendocrine pathways. Altered circadian signaling interferes with the normal function of the hypothalamic-pituitary-thymic axis, resulting in diminished melatonin production or loss of its rhythmic secretion pattern, which in turn weakens natural killer cell activity and cytotoxic T-lymphocyte responses (48). Concurrently, decreased melatonin levels may indirectly enhance sympathetic tone, fostering a pro-tumorigenic neuroendocrine milieu (49, 50).

As a key circadian effector, melatonin has attracted considerable interest for its antitumor properties in BC. It inhibits the p38 MAPK-MMP2/MMP9 axis, interferes with the Akt/GSK3β/β-catenin pathway, and suppresses tumor invasion and metastasis, thereby reducing epithelial-mesenchymal transition and tumor stemness (51). It has also been reported that it can enhance chemosensitivity (52). At the same time, melatonin affects the tumor microenvironment by regulating immune function. Reduced melatonin levels are associated with weakened natural killer cell activity and impaired cytotoxic T cell responses. Moreover, in cases of circadian rhythm disruption, changes in melatonin secretion may affect sympathetic nerve tension and a broader neuroendocrine balance. Overall, circadian disruption should be viewed not merely as a consequence of adverse lifestyle patterns, but as a systemic disturbance of the neuroendocrine-immune network that actively promotes immune escape and BC progression (53), thereby offering a conceptual basis for the development of chronobiology-informed therapeutic strategies.

### 2.5 Clinical perspectives: novel adjuvant strategies in BC therapy

#### 2.5.1 β-adrenergic blockers: repurposing cardiovascular agents for immunomodulatory cancer therapy

Evidence implicating the sympathetic nervous system activity in BC progression has led to growing interest in β-adrenergic receptor antagonists as potential agents for drug repurposing. Within this category, propranolol, a non-selective β-blocker, is supported by the most extensive experimental and clinical data.

Both *in vitro* and *in vivo* studies show that propranolol suppresses epithelial–mesenchymal transition, reduces the expression of mesenchymal-associated genes, and limits tumor cell migration and invasion (54), resulting in decreased distant metastatic spread, particularly to the lungs (55). These effects are closely linked to blockade of β-adrenergic signaling and interference with stress-induced tumor-promoting pathways.

From a clinical standpoint, retrospective analyses indicate that BC patients treated with propranolol at diagnosis or during therapy tend to present with lower tumor grade and experience significantly reduced BC-specific mortality after adjustment for confounding variables (56). Such observations imply that β-blockers may provide adjunctive antitumor benefits, not only by directly affecting cancer cell behavior but also through reshaping immune interactions within the tumor microenvironment.

Although propranolol is the most widely used beta receptor blocker in BC research, other β-adrenergic antagonists have also been evaluated for their effects on BC. Clinical observations suggest that the potential anti-tumor benefits may vary depending on receptor selectivity. Non-selective β-receptor blockers (which can inhibit both beta1 and β2 adrenergic receptors) are more consistently associated with reduced metastasis rates and improved survival rates (57), while there is currently no conclusive evidence for β1-selective drugs (such as atenolol or metoprolol). This difference may reflect the dominant role of β2 adrenergic signaling in tumor progression and immune suppression. However, the existing data are mostly retrospective and diverse, and direct comparative studies between different classes of beta receptor blockers are still limited.

Notably, methods that target tumor-associated sympathetic nerves or globally reduce sympathetic activity may be more effective than selective receptor blockade in reducing immune checkpoint molecule expression, including PD-1, PD-L1, and FOXP3 (58). This observation further supports the role of β-adrenergic signaling as a central nexus linking neural regulation to immune evasion in BC therapy. The therapeutic potential of combining neural-targeted interventions with immunotherapy is further exemplified by advances in other solid tumors, where antibody-drug conjugates designed to induce immunogenic cell death are being rationally combined with checkpoint inhibitors to achieve synergistic antitumor effects (59). Such combinatorial paradigms may inform future clinical trial design in BC, integrating β-blockers or other neuroregulatory agents with established immunotherapies.

#### 2.5.2 Integration of psychological interventions and neuroregulation: a multidisciplinary approach to improving immune prognosis

Apart from drug intervention, the regulation of psychological stress is also an indispensable part of the neuro-immune regulation. During the diagnosis and treatment of BC, patients are often exposed to various psychological stress states for a long time, such as persistent anxiety or depression. Such stress is not a short-term reaction but may maintain a high activity state of the sympathetic nervous system and the hypothalamic-pituitary-adrenal axis for a long time, thereby weakening the anti-tumor immune response at the systemic level.

Chronic psychological stress impairs intratumoral CD8^+^ T cell function, promotes immunosuppressive microenvironment formation, and accelerates disease progression, in part through alterations in gut microbiota composition (34). Conversely, interventions targeting psychological stress—including counseling, mindfulness-based practices, and stress management programs—may indirectly enhance antitumor immune responses by reducing sympathetic excitability and restoring neuroendocrine balance (60). In recent years, randomized controlled trials in the fields of gastric cancer and rectal cancer have further suggested that such interventions may have positive effects on disease-free survival and overall survival (61, 62), but these findings still require larger-scale studies for confirmation. In BC patients, there is currently a lack of direct evidence on whether psychological intervention can translate into long-term oncological benefits.

Accordingly, incorporating psychological interventions into comprehensive BC management, alongside pharmacotherapy and immunotherapy, holds promise for improving immune competence, delaying tumor progression, and reducing recurrence risk. This neuroregulation-centered, multidisciplinary paradigm offers novel clinical perspectives for adjuvant BC treatment.

### 2.6 Summary and outlook

#### 2.6.1 Toward precision medicine: targeting neural plasticity and heterogeneity in BC

Available data indicate that sympathetic nerve activity in BC is generally associated with a dampening of both innate and adaptive immune responses, a state that may facilitate immune escape and support tumor growth. In comparison, the involvement of sensory and parasympathetic nerves is less uniform. Depending on disease stage and local microenvironmental conditions, these neural inputs have been reported to exert either pro-tumor or inhibitory influences on tumor behavior.

These observations underscore the considerable heterogeneity and plasticity of neural regulation in BC. Next, the research focus should be on systematically depicting the neural distribution characteristics within BC tumors, and further clarifying the specific mechanisms of action of different neural subtypes in tumor progression and immune regulation. Special attention should be paid to the combination of neuroregulatory strategies and immunotherapy. Clinical research suggests that the intervention of β-adrenergic signaling may help enhance the anti-tumor immune response, thereby improving the efficacy of immune checkpoint inhibitors. Therefore, combining neuroregulatory intervention with immunotherapy represents a promising therapeutic direction and is worthy of prospective clinical evaluation. At the same time, it will be important to assess neuroregulatory strategies in combination with established treatments, such as immunotherapy and chemotherapy, to better define their potential synergistic benefits as well as safety profiles. A clearer separation between tumor-associated nerves and normal neural structures, together with the discovery of reliable nerve-related biomarkers, could open new avenues for therapies that act on both malignant cells and their neural niche. Such advances highlight the relevance of neural components in BC management. Moreover, drawing on methodological advances from other malignancies, such as the web-based prognostic nomogram developed for elderly patients with primary colorectal lymphoma (63), integrating clinical, neural, and immune signatures into a comprehensive prediction tool could enable more precise risk stratification and personalized treatment in BC.
